# Histone Methyltransferase DOT1L as a Promising Epigenetic Target for Treatment of Solid Tumors

**DOI:** 10.3389/fgene.2022.864612

**Published:** 2022-04-13

**Authors:** Elena Alexandrova, Annamaria Salvati, Giovanni Pecoraro, Jessica Lamberti, Viola Melone, Assunta Sellitto, Francesca Rizzo, Giorgio Giurato, Roberta Tarallo, Giovanni Nassa, Alessandro Weisz

**Affiliations:** ^1^ Laboratory of Molecular Medicine and Genomics, Department of Medicine, Surgery and Dentistry “Scuola Medica Salernitana”, University of Salerno, Baronissi, Italy; ^2^ Medical Genomics Program and Division of Oncology, AOU “S. Giovanni di Dio e Ruggi d’Aragona”, University of Salerno, Salerno, Italy; ^3^ Genome Research Center for Health—CRGS, Campus of Medicine of the University of Salerno, Baronissi, Italy

**Keywords:** targeted cancer therapy, small-molecule inhibitor, histone methyltransferase, epidrug, cancer biomarker

## Abstract

The histone lysine methyltransferase DOT1L (DOT1-like histone lysine methyltransferase) is responsible for the epigenetic regulation of gene expression through specific methylation of lysine79 residue of histone H3 (H3K79) in actively transcribed genes. Its normal activity is crucial for embryonic development and adult tissues functions, whereas its aberrant functioning is known to contribute to leukemogenesis. DOT1L is the only lysine methyltransferase that does not contain a SET domain, which is a feature that allowed the development of selective DOT1L inhibitors that are currently investigated in Phase I clinical trials for cancer treatment. Recently, abnormal expression of this enzyme has been associated with poor survival and increased aggressiveness of several solid tumors. In this review evidences of aberrant DOT1L expression and activity in breast, ovarian, prostate, colon, and other solid tumors, and its relationships with biological and clinical behavior of the disease and response to therapies, are summarized. Current knowledge of the structural basis of DOT1L ability to regulate cell proliferation, invasion, plasticity and stemness, cell cycle progression, cell-to-cell signaling, epithelial-to-mesenchymal transition, and chemoresistance, through cooperation with several molecular partners including noncoding RNAs, is also reviewed. Finally, available options for the treatment of therapeutically challenging solid tumors by targeting DOT1L are discussed.

## Introduction

Histone modifications play a pivotal role in the epigenetic regulation of gene expression, controlling chromatin remodeling and establishment/maintenance of euchromatin and heterochromatin equilibrium. The involvement of modified histones in the pathogenesis of several diseases, in particular cancer, and their reversible nature provides opportunities for the development of specific therapeutic strategies targeting the enzymes involved and the pathways that control their activity (Allis and Jenuwein, 2016). The main chemical histone modifications are represented by methylation, acetylation, ubiquitination, SUMOylation, and phosphorylation, and the enzymes controlling these modifications are classified as “writers” and “erasers,” whereas “readers” are the nuclear factors able to recognize them (Biswas and Rao, 2018). Histone methylation, catalyzed by histone methyltransferases (HMTs), is one of the most studied epigenetic modifications, known to be involved in cell differentiation, modulation of gene expression, DNA recombination, and damage repair (Copeland et al., 2009; Murn and Shi, 2017). This epigenetic modification is controlled by histone lysine methyltransferases (HKMTs) and protein arginine methyltransferases (PRMTs), which catalyze the addition of a methyl group in lysine and arginine residues, respectively.

Aberrant histone hypermethylation has been associated with the development of multiple cancers (Li et al., 2014; Tran et al., 2017; Wilson et al., 2017; Chen et al., 2018), and HMT inhibitors represent a promising therapeutic option for the treatment of these diseases. However, the presence of highly homologous HMTs often results in nonspecific and off-target effects of inhibitors (Zucconi and Cole, 2017). This poor specificity is a consequence of the fact that methylation by the majority of HMTs is catalyzed by SET domains, which transfer a methyl group from S-adenosyl L-methionine (SAM) to a specific amino acid residue of the histone substrate since many inhibitors of HMT SET domains represent SAM analogs built upon or derived from the SAM scaffold. The only HMT that does not contain the SET domain is DOT1L (DOT1-like histone lysine methyltransferase), which is an evolutionarily conserved histone methyltransferase that plays a critical role in modulation, target gene expression by mono-, di-, and tri-methylation of the lysine 79 of histone H3 (H3K79), a key epigenetic modification controlling chromatin remodeling (Nguyen and Zhang, 2011). DOT1L is involved in the modulation of a wide range of biological processes such as telomeric silencing (Singer et al., 1998), DNA repair (Huyen et al., 2004), transcription elongation (Mueller et al., 2007), cell cycle regulation (Jones et al., 2008), cell development (Barry et al., 2009), and immune response (Kealy et al., 2020). Initially, DOT1L was associated with cancer progression following the discovery of its crucial role in the development of mixed lineage leukemia (MLL), where the activity of this enzyme has been extensively studied for the last 20 years. MLL-rearranged (MLLr) leukemia is one of the most aggressive blood tumors, generally characterized by chromosomal translocations of *MLL* gene, located on chromosome 11q23 (Ziemin-Van Der Poel et al., 1991; Djabali et al., 1992; Krivtsov and Armstrong, 2007). In normal cells, MLL protein is targeted by specific cofactors and DNA sequences at specific chromosomal loci, where it catalyzes lysine 4 methylation in histone H3 (H3K4), leading to transcriptional activation of nearby genes (Ayton and Cleary, 2001). Genomic rearrangements induce in MLL loss of the SET domain, responsible for H3K4 methylation, and its fusion to one of 50 different proteins, including the members of the AF and ENL protein families, such as AF4, AF9, AF10, and ENL (Banday et al., 2020). MLL-fused proteins lack the ability to methylate H3K4, but retain the DNA-binding domain of MLL, therefore causing aberrant regulation of MLL target genes, including upregulation of *HOXA7* and *HOXA9* genes, whose alteration is considered a hallmark of MLLr-mediated transformation (Ayton and Cleary, 2001; Hess, 2004; Farooq et al., 2016). However, it was observed that despite absence of an SET domain, MLL-fused proteins somehow retain the ability to increase lysine methylation in target genes. This paradox was explained when some of these chimeric proteins were shown to bind DOT1L, leading to H3K79 methylation and transcriptional activation of MLL target genes, including *HOX* family genes (Okada et al., 2005; Bernt et al., 2011). Noteworthy, DOT1L protein, although strongly involved in leukemogenesis, was never found mutated, suggesting that the driver of neoplastic transformation is linked in these cases to aberrant positioning of this enzyme in the genome (Okada et al., 2005; Bernt et al., 2011). These findings provided the rationale for proposing that DOT1L may represent an actionable target for the treatment of these leukemias.

DOT1L activity in cancer cells is controlled at multiple levels starting from the regulation of the methyltransferase expression to context-dependent modulation of its enzymatic performance. DOT1L expression is known to be regulated by transcription factors (Wong et al., 2017), small noncoding RNAs (Lv et al., 2019), and, posttranslationally, through its acetylation (Liu et al., 2020) or O-GlcNAcylation (Song et al., 2021). This contributes to protein stability allowing the enzyme to escape from the ubiquitination and consequent proteasome degradation. An additional mechanism of fine-tuning of DOT1L activity is achieved by its cofactor-mediated recruitment to specific genomic locations. The known examples of this type of regulation are the transcription factor-mediated DOT1L recruitment to promoter regions of target genes (Cho et al., 2015; Nassa et al., 2019; Yi et al., 2020) and the stimulation of its activity by binding to mono-ubiquitinated histone H2B (Valencia-Sánchez et al., 2019; Spangler et al., 2022) and histone H4 tail that result in the precise positioning of the methyltransferase catalytic center above H3K79 (Worden et al., 2019).

Recently, the DOT1L gene has been found overexpressed and/or amplified in certain nonhematologic malignancies, where the prognostic and pathogenetic significance of an aberrant expression of this enzyme has been characterized. This review summarizes the available information concerning these evidences and the known roles of DOT1L in solid tumors, including its molecular and genomic actions, and presents a rationale for considering DOT1L as a novel therapeutic target also against these malignancies.

## DOT1 Prognostic Potential in Solid Tumors

**FIGURE 1:**
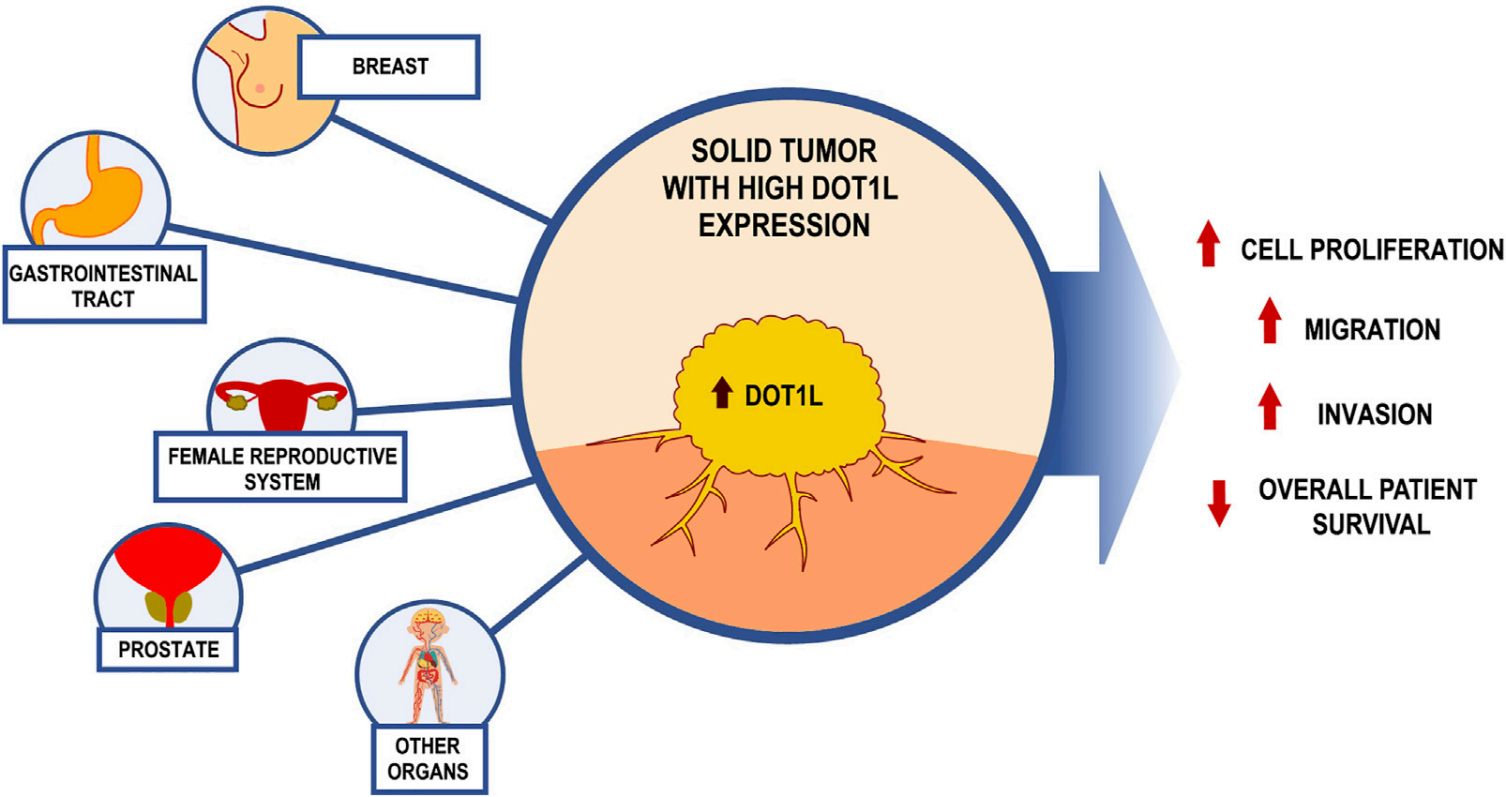
Consequences of excessive DOT1L levels on the malignant phenotype of solid tumors.

High DOT1L expression has been found in a number of nonhematologic neoplasms including breast (Lee and Kong, 2015; Nassa et al., 2019), prostate (Annala et al., 2014), ovarian, gastric cancer (Wang X. et al., 2019), and other malignancies (Figure 1).

In the mammary gland, DOT1L promotes multistep carcinogenesis, as its expression is required for the malignant transformation of breast epithelial cells and for tumor initiation. Its activity has been associated with poor overall survival and increased tumor aggressiveness, through the enhancement of the metastatic potential of cancer cells (Cho et al., 2015). Interestingly, a recent work suggested an unexpected use of DOT1L as an indicator of eligibility for immunotherapy, as its expression level has been reported to mark immune-activated breast cancers (Noblejas-López et al., 2019). Moreover, DOT1L expression was found to be particularly elevated in estrogen receptor α (ERα)-positive breast tumors, where this associated with a worse clinical outcome (Zhang et al., 2014; Cho et al., 2015). Of note, DOT1L has been found upregulated in breast cancer stem cells (CSC), suggesting that this enzyme plays a critical role in breast tumor initiation promoting tumorigenic and self-renewal activity of CSCs populations (Cho et al., 2015), a finding also confirmed by Oktyabri et al., who elucidated an important role of lysine methyltransferase in determining the stem cell-like properties of breast cancer cells regulating the expression of BCAT1, an enzyme involved in the amino-acid metabolism (Oktyabri et al., 2016). These findings are in line with the results of another in human head and neck squamous cell carcinoma, where it has been demonstrated that DOT1L expression is upregulated by hyaluronan (HA) matrix in a subpopulation of CSCs (Bourguignon et al., 2016).

Interestingly, high DOT1L expression has been shown to represent a biomarker of poor prognosis also in other gynecological malignancies, in particular ovarian cancer (Zhang et al., 2017), where the expression of DOT1L has been associated with chemoresistance (Liu D. et al., 2018) and shorter relapse-free (RFS) and overall (OS) survival (Alexandrova et al., 2020; Chava et al., 2021). In a recent work by Cheasley et al., targeted sequencing identified DOT1L among putative driver genes mutated in low-grade serous ovarian carcinoma (LGSOC), which is characterized by a poor response to chemotherapy (Cheasley et al., 2021). In line with these findings, a cooperative action of DOT1L with the transcription factor C/EBPβ, which represents a prognostic factor in ovarian cancer, is involved in increased chemoresistance of tumor cells (Liu D. et al., 2018). On the other hand, a controversial result from the *in vitro* CRISPR/Cas9 study of Wang et al. points to a potential tumor suppressor role of this enzyme in OC (Wang X. et al., 2019), although this finding has not been confirmed yet. Furthermore, DOT1L activity was reported as a crucial risk factor for the development of cervical cancer associated with the human papilloma virus (HPV) infection (Liu Y. et al., 2018). The employment of epigenetic drug targeting HMTs represents an interesting therapeutic opportunity also in endometrial cancer (Inoue et al., 2021.), but the usefulness of DOT1L inhibition against these tumors still remains to be clarified.

In males, marked implication of DOT1L has been shown in prostate cancer (PCa), where the over-expression of this methyltransferase is an indicator of poor outcome. Interestingly, activity of these enzyme results strongly linked the tumorigenic potential of androgen receptor (AR)-expressing cancer cells (Vatapalli et al., 2020), in line with the known involvement of this HMT in AR signaling in prostate cancer cells (Yang et al., 2013).

Involvement of DOT1L in gastric cancer has been well elucidated by a recent work, demonstrating a direct correlation between the level of this gene mRNA and tumor differentiation grade, TNM staging, and lymph node metastases, providing a clinical demonstration that DOT1L expression may represent an independent prognostic marker of OS in gastric cancer patients (Song et al., 2020). These results confirmed a previous report by Donner et al. (2015), showing that DOT1L plays a role in hereditary diffuse gastric cancer (HDGC) pathogenesis. In this study, carried out in a small cohort of patients with hereditary gastric cancer identified, a DOT1L variant (p.Pro1146Leu) was identified by exome sequencing, among other candidate susceptibility genes, in affected individuals but not in control cases (Donner et al., 2015).

The link between a higher *DOT1L* expression and a worst patient’s prognosis has been confirmed also in other malignancies of the gastrointestinal tract, such as in colorectal cancer (CRC), where a positive correlation between *DOT1L* and *MYC* expression was found in several cohorts of CRC patients (Yang et al., 2019). A further indication that this epigenetic regulator may represent a predictor of poor patient survival comes from the observation that DOT1L expression in mice is prompted by the stimulation of cancer cells with interleukin-22 (IL-22), predominantly produced by CD4^+^ T cells, resulting in enhanced CRC cell stemness (Kryczek et al., 2014). Interestingly, it has been recently demonstrated how the expression of DOT1L can be regulated by small noncoding RNAs, such as miR-133b, a microRNA with a tumor suppressor role, whose activity determines the downregulation of DOT1L at both mRNA and protein level, regulating CRC stemness and chemoresistance (Lv et al., 2019). In addition, in line with the association between DOT1L and MYC, an elegant study (Wong et al., 2017) demonstrated that this lysine methyltransferase is involved in neuroblastoma oncogenesis, and its mRNA and protein expression upregulated by N-Myc.

In line with these findings, DOT1L expression represents an indicator of poor OS, RFS, and postoperative recurrence also in patients with clear-cell renal cell carcinoma (ccRCC), as demonstrated by a recent retrospective study that enrolled a large cohort of 282 patients (Qu et al., 2016).

Finally, DOT1L expression has been correlated with poor patient survival also in human neuroblastoma, a finding confirmed also in mice xenografts, where DOT1L ablation significantly reduced neuroblastoma tumor growth and improved OS (Wong et al., 2017).

The heterogenous nature of the cancer types listed above suggests a context-independent involvement of DOT1L in development and progression of multiple solid neoplasms.

## Mechanisms of DOT1 Action in Solid Tumors

### Breast Cancer

Breast cancer (BC) is the most common cancer type and the second leading cause of cancer-related death in women worldwide. BC is generally classified into three main subtypes, based on expression of estrogen receptors (ERs), progesterone receptors (PRs), and/or human epidermal growth factor 2 (HER2). This categorization guides therapeutic decisions, as patients expressing hormone receptors are mainly subjected to endocrine therapy (ET), whereas HER2-positive tumors represent best candidates for targeted and immune therapies. Triple-negative breast cancer (TNBC), instead, is the most aggressive mammary tumor subtype and is characterized by the absence of these three molecular markers and, to date, limited therapeutic options are available against these diseases (Waks and Winer, 2019).

Recently, epigenetic modifications have been suggested as key factors of breast carcinogenesis (Garcia-Martinez et al., 2021). In particular, the enzymes controlling the main histone marks, known as epigenetic “writers” (e.g., DOT1L) and ‘”erasers,” together with the epigenetic “readers” are able to modulate the transcriptional programs underlying breast carcinogenesis, tumor growth, and progression. The reversibility of the modifications catalyzed by these epi-enzymes, and the discovery of several inhibitors of their activity, gave rise to investigations focusing on the use of epi-enzymes as molecular targets for BC treatment. Moreover, novel approaches based on combinatorial epigenetic therapy (epi-drug) with chemotherapies, ET, or immunotherapy may represent new therapeutic strategies to overcome tumor relapse exploitable for BC clinical management (Buocikova et al., 2020).

A role of DOT1L in the cellular events controlling BC aggressiveness has been demonstrated in a study of Duan *et al.*, where aberrant DOT1L activity was associated with the metastatic potential of BC cells (Duan et al., 2019). This result, together with the direct correlations demonstrated between high DOT1L levels in tumors, poor disease prognosis, and worse overall and relapse-free survival (Cho et al., 2015; Nassa et al., 2019), suggests the importance of this enzyme in BC and its eligibility as a promising therapeutic target against these neoplasms.

TNBC represent nearly 20% of mammary tumors, do not respond to hormone or other targeted therapies, and are associated with high metastatic potential and bad prognosis. It was shown that the inhibition of H3K79 methylation reduces proliferation, migration, and invasiveness of TNBC cells, blocking metastatic process (Zhang et al., 2014; Byun et al., 2019). In this contest, DOT1L acts as oncogene, cooperating with c-Myc-p300 transcriptional complex that recruits DOT1L to promoter region of target genes through the DNA-binding ability of c-Myc (Cho et al., 2015). This cooperation catalyzes methylation of histones in target genes such as *SNAIL*, *ZEB1*, and *ZEB2* that, once upregulated, promote epithelial-to-mesenchymal transition (EMT) and metastatic cell diffusion. In addition, the DOT1L activity enhances the neoplastic transformation of CSCs, closely associated with EMT (Tanabe et al., 2020), drug resistance, and high tumor relapse (Cho et al., 2015). Consistent with these findings, DOT1L inhibition by EPZ004777 was found to be effective in increasing E-cadherin levels in BC cells, by repressing some key transcription factors involved in EMT promotion in these cells (Cho et al., 2015). Furthermore, this HMT regulates TNBC cell migration and mammosphere formation by transcriptional regulation of *BCAT1*, encoding an enzyme involved in catabolism of branched chain amino acids and upregulated in several cancers (Oktyabri et al., 2016). Overall, these data suggest the possibility of blocking several modulators of BC aggressiveness by inhibiting this epigenetic writer.

In addition, *ZEB2* and *MALAT1* were found to be modulated by DOT1L also in ERα-positive BC cells, which express the primary oncogenic driver of the majority of BCs. Overexpression of this enzyme led to increased *ZEB2* and *MALAT1* gene transcription, indicating a role of DOT1L in EMT regulation and BC metastasis also in luminal cells (Duan et al., 2019).

The current endocrine-based therapeutic options for ERα-positive BCs include ERα-blockade with antiestrogens, selective ERα degradation with specific inducers, and inhibition of local estrogen synthesis with aromatase inhibitors. Although ET extend overall survival, a third of all early-stage ER+ breast cancer patients experience resistance to these treatments, making it necessary to identify new molecular targets in cancer cells to bypass resistance mechanisms. DOT1L participates in regulation of several functional pathways in hormone-responsive BC, including P53 and MAPK signaling, whose alterations are known to be involved in these tumors (Gregoire et al., 2016). Nassa et al. found DOT1L as a component of ERα cistrome shares with this nuclear receptor several nuclear interactors essential for BC cell survival and proliferation (Nassa et al., 2019; Salvati et al., 2020). Interestingly, this enzyme was also found to interact with antiestrogen (Tamoxifen and ICI)-bound ERα (Cirillo et al., 2013), suggesting that it might be involved also in the residual estrogen-like activity of these drugs. Pharmacological inhibition of DOT1L reduced hormone-responsive and endocrine-resistant BC cell proliferation rate and increased cell death both *in vitro* and *in vivo*, an effect due to the blockade of ERα signaling by epigenetic downregulation of ERα expression mediated by the HMT activity of this factor.

Finally, Noblejas-López et al. identified DOT1L as a neopeptide produced by basal-like BC cells (Noblejas-López et al., 2019). These molecules are expressed in a tumor-specific manner showing high-affinity binding with major histocompatibility complex (MHC). *In silico* analysis identified specific mutation in DOT1L sequence, which shows a high binding affinity for HLA isotype (HLA-A020), whose upregulation is associated with the initiation of immune response and a favorable prognosis in basal-like breast cancers.

### Other Gynecological Cancers

According to GLOBOCAN, in 2020 estimated gynecological cancer incidence comprised >1.350.000 new cases and up to 670.000 deaths (Sung et al., 2021). Uterine (cervix and endometrium) and ovarian carcinomas are the most frequent neoplasms, representing 43.2%, 29.8%, and 22.4% of all gynecological malignancies, responsible for 50.9%, 14.4%, and 30.8% of deaths, respectively (Sung et al., 2021). In the majority of cases, surgery in combination with chemotherapy represents the main therapeutic option, whereas in fewer cases radiotherapy or immunotherapy is applied (Kandalaft et al., 2019; Yang et al., 2020; Dyer et al., 2021).

Ovarian cancer (OC) is an indolent disease, frequently diagnosed at advanced stages due to the lack of specific symptoms, characterized by low response rate to current treatments and a high incidence of induced resistance (Yang et al., 2018). These tumors are highly heterogeneous and classified into several subtypes, each characterized by distinct gene expression, epigenetic and mutational patterns, and, consequently, critically differing one from another. Among OCs, high-grade serous ovarian cancer (HGSOC) represents the most common, aggressive, and lethal form of epithelial cancer (Gifford et al., 2004; Glasspool et al., 2006), showing an urgent need of novel therapeutic targets and treatments. In cervical cancer, the primary risk factor is represented by infection with high-risk human papillomaviruses (HR-HPVs) (Castellsagué, 2008), which through expression of the two oncogenic proteins E6 and E7 orchestrate the molecular pathways leading to cancer initiation and progression.

A role for DOT1L in gynecological malignancies was first proposed by Zhang et al. in a study where prognostic significance of DOT1L in ovarian cancer was evaluated (Zhang et al., 2017). These authors demonstrated that expression of DOT1L protein is significantly increased in malignant ovarian tumors and that a high DOT1L expression correlates with advanced FIGO (Federation of Gynecology and Obstetrics) tumor stage, high histologic grade, and the presence of lymph node metastasis in OC patients. Interestingly, DOT1L was shown to represent an independent prognostic biomarker of OS and PFS in OC, as patients carrying tumors characterized by high DOT1L expression exhibited a significantly reduced overall and progression-free survival (Zhang et al., 2017). These results were further confirmed by another study (Cumhur Cure and Cure, 2021), where using publicly available OC datasets it was demonstrated that DOT1L mRNA expression was significantly higher in OC tumors, compared with normal ovarian tissue, and that high expression of this gene is associated with reduced PFS and OS. In addition, Salvati et al., focusing on HGSOC, the most aggressive and lethal form of OC (Vaughan et al., 2011), showed that OS of patients with high DOT1L expression was significantly impaired in ERα-positive tumors (Salvati et al., 2019).

Several mechanisms of DOT1L action in OC have been described. Zhang *et al.* reported that this methyltransferase is able of direct binding and transcriptional activation of *CDK6* and *CCND3* genes (Zhang et al., 2017), known to be involved in cell cycle G1 phase progression (Sherr, 1995), whereas DOT1L knock-down or pharmacological inhibition induced reduction of H3K79 dimethylation level of these genes, and accumulation of cells in G1 (Zhang et al., 2017). In another study, genetic knockdown of DOT1L, or its pharmacological inhibition by specific inhibitors, was found to significantly impair survival and the tumor-forming potential of multiple OC cell lines both *in vitro* and *in vivo* (Zhang et al., 2017; Chava et al., 2021). Importantly, the growth suppressive effect of DOT1L inhibitors was dependent upon the expression level of the enzyme, because OC cells expressing the highest level of DOT1L and H3K79me2 mark in chromatin ì exhibited the highest sensitivity to the drug (Chava et al., 2021). Moreover, DOT1L blockade induced apoptosis and necrosis of OC cells (Chava et al., 2021), due to upregulation of genes involved in cell death signaling/apoptotic pathways and downregulation of cellular biosynthesis pathways including amino acid and nucleotide metabolic pathways. This result was confirmed also by cell death evaluation and mass spectrometry-mediated analysis of metabolome changes, respectively (Chava et al., 2021). Of note, treatment of high DOT1L-expressing OC cells with specific inhibitors induced also significant upregulation of the NKG2D ligand ULBP1 and enhanced NK cell-mediated killing of the OC cells (Chava et al., 2021).

In support of this possibility, Salvati and colleagues demonstrated that pharmacological blockade of DOT1L exerts an antiproliferative effect mediated by cell cycle inhibition and loss of colony formation capability also in ERα-positive HGSOC cells (Salvati et al., 2019). Comparison of transcriptome profile changes induced by antiestrogen-mediated ERα blockade and DOT1L inhibition revealed the presence of a subset of genes, commonly modulated by the two conditions, involved in invasiveness/migration and control of key signaling cascades, suggesting a functional cooperation between ERα and DOT1L in these cells (Salvati et al., 2019). Furthermore, DOT1L was found to be associated with ERα on chromatin, where the two proteins share several common targets. Interestingly, DOT1L inhibition evoked a dose-dependent reduction of ERα expression caused by reduced DOT1L binding to the promoter region of the ERα gene, abolishment of H3K79me2 and H3K4me3 active transcription marks, and accumulation of H3K27me3, associated with repressive chromatin state. In line with these findings, co-inhibition of ERα and DOT1L has a synergetic effect on the proliferation of OC cells, including subclones resistant to platinum derivatives (Salvati et al., 2019).

Liu *et al.* found that DOT1L is recruited to multiple drug-resistance genes in OC cells, through cooperation with the transcription factor C/EBPβ, where it maintains an open chromatin state by H3K79 methylation, conferring in this way chemoresistance to OC cells. In practice, direct interaction and co-occupancy of the same gene loci was shown for these two proteins. In addition, C/EBPβ-DOT1L common targets including genes involved in cisplatin resistance *via* drug transport, DNA damage repair, and cell survival (Liu D. et al., 2018).

In apparent contrast with the results reported above, Wang et al. reported that complete loss of DOT1L expression in OC promotes cell invasion and stemness through the downregulation of E-cadherin and TJP1 and the upregulation of ALDH1A1 expression by Wnt signaling activation, suggesting that this enzyme could exert also tumor suppressive role in certain OC cells types (Wang X. et al., 2019).

When combined, these data strongly suggest the need to further investigate the roles of DOT1L in OCs to exploit the available inhibitors of this HMT to devise new protocols to identify affective therapeutic regimens against these tumors.

DOT1L involvement in cervical cancers associated with persistent high-risk human papilloma virus (HR-HPV) infection has been recently demonstrated. In a study by Liu et al., the E7 viral protein was found to induce increased intracellular reactive oxygen species (ROSs) that, in turn, triggers translocation of the key glycolysis enzyme Lactate Dehydrogenase A (LDHA) from cytoplasm to the nucleus, where it acquires a noncanonical activity leading to the production of an antioxidant metabolite α-hydroxybutyrate (α-HB) (Liu Y. et al., 2018). α-HB accumulation induced H3K79 hypermethylation promoting the interaction between DOT1L and LDHA and, thereby, activation of DOT1L enzymatic activity by an unknown mechanism. Further analysis demonstrated that oxidative stress enhanced H3K79 di-methylation and overexpression of antioxidant genes *SOD1* and *CAT*, and Wnt target genes *CTNNB1* and *MYC*. Genetic ablation of LDHA blocked ROS-induced H3K79 hypermethylation in these four genes, while depletion of transcription factor NRF2, known to be involved in cellular redox sensing (Sporn and Liby, 2012), induced similar effects only on H3K79 di-methylation of *SOD1* and *CAT* genes, indicating DOT1L involvement in the regulation of cervical cancer cell survival and proliferation in conditions of oxidative stress, *via* activation of Wnt signaling and redox balance control through a LDHA-DOT1L-NRF2 axis (Liu Y. et al., 2018).

Altogether, these data indicate that in gynecologic cancers DOT1L may act via the regulation of expression of genes involved in cell cycle progression, cell death, EMT, stemness, metabolism, oxidative stress response, and drug-resistance through direct binding to transcriptional factors or other cofactors, and might represent a potential prognostic biomarker and potential therapeutic target.

### Prostate Cancer

DOT1L results in an actionable and promising therapeutic target also for PCa, although its role in prostate cells is still poorly defined (Kgatle et al., 2016; Vatapalli et al., 2020). PCa is one of the most common and lethal malignancies affecting men worldwide (Miller et al., 2019; Rebello et al., 2021); every year, more than 1.2 million new cases and 350,000 deaths are recorded (Bray et al., 2018). The main factor driving PCa development and progression is the nuclear receptor AR (Shen and Balk, 2009). This transcription factor, following activation by its cognate hormone ligands testosterone and dihydrotestosterone, translocates into the nucleus where it binds androgen-responsive elements (AREs), regulatory genetic elements on DNA, and thereby drives transcriptome changes involved in cell proliferation and differentiation (Heinlein and Chang, 2004; Shen and Balk, 2009). In cancerous cells, the mitogenic activity of AR is considered a key event for the maintenance of the transformed status and for tumor expansion as well as progression, and for this reason the standard treatment for AR-positive PCas includes androgen deprivation therapy (ADT), which targets the AR signaling pathway (Shen and Balk, 2009). Unfortunately, most patients develop resistance to this pharmacological regimen, leading to the development of an aggressive tumor phenotype known as castration-resistant/recurrent PCa (CRPC) (Shen and Balk, 2009). This is driven, in the majority of cases, by AR gene alterations, including amplification and mutations (Karantanos et al., 2015). Novel therapeutic approaches are required in these cases to enhance and improve current therapeutic options. The role of DOT1L as a potential therapeutic target in PCa and its involvement in AR signaling was recently evaluated (Yang et al., 2013; Vatapalli et al., 2020). Analysis of different datasets revealed the upregulation of *DOT1L* expression in PCa, compared with the normal prostate, associated also with poor disease-free survival associated with high expression of this HMT (Vatapalli et al., 2020). These authors demonstrated that DOT1L inhibition selectively regulates the tumorigenicity of AR+ PCa cells and organoids, including CRPC cells (Vatapalli et al., 2020). In addition to the loss in colony formation and cell viability, treatment with a specific inhibitor caused decrease in both AR and MYC protein levels in these cells. The authors hypothesized that a distal K79 methylation-marked enhancer is involved in the regulation of *MYC* gene expression by AR and DOT1L, since both these two proteins were found associated with this gene promoter in AR+, but not in AR-, cells. Furthermore, reduction of MYC expression, induced by DOT1L inhibition, leads the upregulation of MYC-regulated E3 ubiquitin ligases, HECTD4 and MYCBP2, that in turn promoted the degradation of both AR and MYC (Vatapalli et al., 2020).

DOT1L was also described to be a part of a mechanistic link between PCa-upregulated lncRNAs, PRNCR1, PCGEM1, and AR transcriptional activity, an event occurring also in PCa-resistant cells (Yang et al., 2013). According to this mechanism, PRNCR1 binds at the carboxy-terminally acetylated AR on enhancers and its association with DOT1L appears to be necessary for the recruitment of PCGEM1, the second lncRNA, to the AR amino terminus that is methylated in its K349 residue by DOT1L (Yang et al., 2013). This mechanism could explain why DOT1L knockdown affected AR interaction with PCGEM1, but not with PRNCR1, since AR methylation, mediated by PRNCR1-bound DOT1L, is critical for the recruitment of PCGEM1 to AR (Yang et al., 2013). According to these results, these lncRNAs, overexpressed in PCa, can interact with truncated and full-length AR, causing ligand-independent activation of the AR transcriptional program and cell proliferation (Yang et al., 2013).

A study of Annala et al. also reported DOT1L involvement in prostate carcinogenesis. In particular, they showed that growth of AR-negative PCa may be driven by a *DOT1L-HES6* fusion gene, originating by an interchromosomal rearrangement that fused intron 9 of DOT1L with a position 4 kb upstream of *HES6*, resulting in overexpression and pathological activation of this gene, encoding a transcription factor representing a key player in the induction of castration-resistant PCa (Annala et al., 2014).

### Gastrointestinal Cancers

Gastrointestinal (GI) cancers represent a group of heterogeneous malignancies that affect the intestines and accessory organs of digestion (Katona and Lynch, 2018). Among GI cancers, esophageal, gastric, and colorectal cancers are the most frequently diagnosed, representing one of the leading causes of GI cancer-related deaths. Despite distinct onset and molecular pathogenesis, all GI cancers are characterized by high lethality, mainly due to lack of early detection methods and a high metastatic potential. As a consequence, new reliable biomarkers and druggable targets are required here to improve diagnosis and clinical management of these diseases (Link and Goel, 2013).

Recently, several studies explored the activity of the histone methyltransferase DOT1L in GI cancers, elucidating its role in tumor growth and response to current treatments.

Two recent studies pointed to DOT1L as a promising target for gynecological cancer (GC) treatment. Specifically, Song et al. (2020) reported that DOT1L knockdown or its pharmacological inhibition causes a cell cycle arrest at G1, reducing cell proliferation of GC cells *in vitro*. Moreover, in mice xenografts, derived from SGC7901 cells, treatment with a specific inhibitor of this enzyme induced reduction of H3K79 di-methylation (H3K79me2), expression of CDK4 and CDK6, cyclin-dependent kinases involved in the regulation of G1 to S transition, and tumor size, suggesting that DOT1L promote cell cycle progression by H3K79 di-methylation in *CDK4* and *CDK6* genes (Song et al., 2020). Using another GC cell model, Wang et al*.* showed that DOT1L knockdown inhibits migration and invasion of GC cells *in vitro* and their ability to form metastasis *in vivo*. The same results were obtained also by *in vitro* inhibition of DOT1L activity by hesperetin treatment, showing also that this enzyme regulates the expression of genes involved in the EMT, in particular the metalloproteinase genes *MMP2* and *MMP9* and those encoding N-cadherin and fibronectin through their H3K79 di-methylation (Wang et al., 2021).

In colorectal cancer (CRC), several findings associated DOT1L and its histone modifications with crucial steps of carcinogenesis, including transcriptional regulation of tumor suppressor genes (TSGs) (Jacinto et al., 2009), stemness (Kryczek et al., 2014; Lv et al., 2019), Wnt signaling pathway (Mahmoudi et al., 2010; Gibbons et al., 2015), cell cycle progression (Yang et al., 2019), tumor invasiveness (Liu et al., 2020), and DNA damage response (Kari et al., 2019) genes.

An oncosuppressive role of DOT1L was proposed in CRC by Jacinto and colleagues that analyzed the epigenetic mechanisms underlying the transcriptional status of TSGs and found a correlation between H3K79me2 levels in these genes and their CpG island methylation status. In particular, they showed how hypermethylation of TSGs promoter regions is followed by the loss of H3K79me2, leading to their suppression (Jacinto et al., 2009). Another study, however, reported that DOT1L increases CRC tumorigenic potential by enhancing cancer stemness programs. Using primary colon cancer cell models, Kryczek et al. showed in fact that DOT1L activates transcription of stemness genes, such as *NANOG*, *SOX2*, and *Pou5F1*, mediating their H3K79 di-methylation (Kryczek et al., 2014). A subsequent work confirmed these observations using CRC cell lines and spheroids, reporting that targeting DOT1L by miR-133b reduces CRC cell chemoresistance and stemness by suppression of H3K79 di-methylation of stem cells genes (Lv et al., 2019). Apparent contrasting evidences were reported concerning DOT1L involvement in the regulation of the Wnt signaling pathway, known to play a crucial role in stemness maintenance and cell proliferation and to be deregulated in cancer (Clevers, 2006). For example, Mahmoudi and colleagues showed that, in cooperation with the leukemia-associated Mllt10/Af10, *via* H3K79 methylation DOT1L positively regulates the RNA elongation step during transcription of Wnt target genes (Mahmoudi et al., 2010). On the contrary, the results obtained by Gibbons et al. suggest the absence of a direct connection between H3K79 methylation and expression of Wnt signaling pathway genes (Gibbons et al., 2015).

In another two studies, an oncogenic activity of DOT1L in GI cancer was described. Yang et al. showed that chamical inhibition or silencing of DOT1L causes cell cycle arrests in S phase, downregulates *c-Myc* transcription, inhibits cell proliferation *in vitro*, and suppresses tumorigenicity *in vivo* (Yang et al., 2019). Confirming these results, Liu and colleagues showed that DOT1L acetylation at K358 positively correlated with CRC cells capability to migrate and form metastases *in vivo*. Indeed, the acetylation mediated by CREB-binding protein (CBP) prevents DOT1L proteosomal degradation by ubiquitination, thereby promoting transcription of genes involved in EMT *via* H3K79 methylation (Liu et al., 2020). In disagreement with these observations, an oncosuppressive role of DOT1L was observed instead in the rectal cancer cell line SW837. Kari *et al.* demonstrated that DOT1L plays a crucial role in homologous recombination (HR), which drives DNA double-stranded breaks (DSBs) repair in the DNA damage response. Indeed, phosphorylation of H2AX at serine 139, needed for the recruitment of DNA repair proteins at DNA damage sites, is decreased in cells treated with DOT1L inhibitors. In addition, the same authors reported a significant correlation between high levels of DOT1L and H3K79me3 and increased OS in a cohort of 156 rectal cancer patients (Kari et al., 2019).

### Other Solid Tumors

Aberrant DOT1L expression or enzyme activity has been found also in other non-hematologic cancers, including lung, melanoma, neuroblastoma, liver, and head and neck squamous cell carcinomas (HNSCCs).

In lung adenocarcinomas, DOT1L has been recently found mutated in 3% of cases analyzed, pointing to excessive H3K79me in a subset of samples (Campbell et al., 2016). In another study, DOT1L downregulation was shown to induce a nonproliferating multinucleated phenotype, with abnormalities in mitotic spindle formation and centrosome number in lung cancer cells, suggesting chromosome mis-segregation (Kim et al., 2012). The chromosomal instability in DOT1L-depleted cells is accompanied by cell cycle arrest in G1 by transcriptional upregulation of cyclin-dependent kinase inhibitors and cell senescence, all phenotypes reversed upon forced expression of a catalytically active DOT1L, but not a kinase-null inactive mutant of the enzyme (Kim et al., 2012). Of note, H3K79 methylation was found upregulated in lung cancer suggesting its association with lung tumorigenesis (Kim et al., 2012), but average *DOT1L* mRNA level in lung tumor tissues results comparable to that of paired normal lung tissues (Kim et al., 2012). Evanno *et al.* found that TGF-β1 causes a significant reduction in DOT1L expression and H3K79me3 levels in lung cancer cells, accompanied by increased EMT (Evanno et al., 2017). DOT1L inhibitors, however, did not induce EMT as expected, but this occurred only when they were combined with the histone deacetylase inhibitor SAHA or the BET bromodomain inhibitor PFI-1. This anti-EMT effect of the HMT was confirmed by its ability to downregulate PD-L1, associated with an inflammatory tumor microenvironment in lung adenocarcinoma (Lou et al., 2016), the cell surface receptor NRP2, and its ligand SEMA3C, both known for their role in EMT induction (Nasarre et al., 2013; Neufeld et al., 2016). In line with these results, Evanno *et al.* reported, under the same condition, an increased expression of the epithelial markers E-cadherin (Evanno et al., 2017). More recently, Serresi and coworkers performed in lung cancer cells a large-scale CRISPR interference (CRISPRi) screen for genes involved in epigenome regulation, to identify factors required for the proper regulation of EMT and found that loss of DOT1L affects epithelial mesenchymal homeostasis, cellular fitness, and migration (Serresi et al., 2021). In this screen, DOT1L deletion significantly impaired EMT, while increasing at the same time cell motility, a result suggesting a complex effect of DOT1L on epithelial/mesenchymal identity and migratory potential in lung cancer (Serresi et al., 2021).

DOT1L has been shown to exert a protective role during UV-induced melanomagenesis, since in the absence of this enzyme UVR-induced DNA damage is inefficiently repaired (Zhu et al., 2018) and missense mutations of *DOT1L* gene were found in 4.4–15% of melanoma cases from publicly available sequence databases, such as The Cancer Genome Atlas (TCGA) and Broad Institute Databases (Hodis et al., 2012; Shain et al., 2015; Zhu et al., 2018). Moreover, a missense R409H germline *DOT1L* mutation has been identified in two members of a family with four melanoma cases, although this mutation does not impair significantly the H3K79 methyltransferase activity of the enzyme (Salgado et al., 2019). Further investigations of the role of DOT1L in melanomagenesis revealed that its inhibition alone does not affect the growth of primary melanocytes or most melanomas, but the presence of activated oncogene BRAF^V600E^ or ultraviolet radiation (UVR) facilitates oncogenic transformation of DOT1L-depleted melanoma cell both *in vitro* and *in vivo* (Zhu et al., 2018). Additionally, it was shown that DOT1L exerts an UVR protective role in melanocytes and its deletion alone is not sufficient to induce melanomagenesis, where concomitant oncogenic events, such as *BRAF* gene mutation, are required. This fact, together with the observation of an increased sensitivity to UV irradiation of cells expressing mutated DOT1L, led to the discovery of an involvement of this enzyme in DNA repair following UVR-induced DNA damage in melanocytes. Indeed, Zhu et al. demonstrated that DOT1L itself and K79me2 H3 directly interact with the nucleotide excision repair (NER) factor XPC in a UVB-dependent manner, thereby inducing efficient XPC recruitment to chromatin in response to UVB-induced DNA damage, providing a template for XPC-mediated NER (Zhu et al., 2018). Along this line, Torre et al. (2021) applied a CRISPR–Cas9 genetic screen in melanoma cells to identify factors involved in the regulation of cellular plasticity targeting their primed state and thereby allowing them to survive BRAF inhibition and to develop drug resistance. Among several factors affecting cell priming, these authors identified DOT1L, whose depletion increased the frequency of primed cells both *in vitro* and *in vivo*. Interestingly, inhibition of DOT1L prior to the addition of a BRAF inhibitor, but not co-administration of both drugs, enhanced resistance of melanoma cells to the latter drugs (Torre et al., 2021).

Recently, high DOT1L expression in neuroblastoma tissues was associated with poor prognosis (Wong et al., 2017), but this was not investigated further so its significance remains unclear. On the other hand, N-Myc was found to regulate DOT1L gene expression directly, by binding to a noncanonical E-box located in the promoter (Wong et al., 2017). These authors found also that DOT1L induces H3K79me2 in the promoter region of the N-Myc target *ODC1* and *E2F2* genes, where in complex with N-Myc itself, it activates the transcription of these genes, confirmed by the finding that depletion or pharmacological inhibition of DOT1L inhibits proliferation of *MYCN*-amplified neuroblastoma cells both *in vitro* and *in vivo*. In another study, Macleod et al. (2019) revealed the ability of DOT1L to regulate stemness and proliferation of glioblastoma stem cells (GSCs). This conclusion was based on the results of a genome-wide CRISPR-Cas9 screening performed in a panel of patient-derived GSCs, further validated *in vitro* by pharmacological inhibition of DOT1L, that induced growth inhibition by G0/G1 cell cycle arrest and apoptosis. Further analyses revealed morphologic changes consistent with differentiation in DOT1L-depleted cells, an observation supported by the decreased expression of stem cell markers, accompanied by reduced GSCs self-renewal, migration, and invasion potential. Finally, DOT1L inhibition induced H3K79me2 loss and consequent downregulation of glioblastoma multiforme fitness genes *SOX2* and *OLIG2*. These data support involvement of this epi-enzyme in the regulation of GSC stemness *in vivo* and suggest potential beneficial effects of DOT1L inhibition for glioblastoma treatment (Macleod et al., 2019).

In head and neck squamous cell carcinoma (HNSCC), DOT1L involvement in regulation of cancer stem cell properties was recently demonstrated. It was shown that a subpopulation of HNSCC CSC-like cells is characterized by high expression of isoform 3 of the cell surface receptor CD44 (CD44v3) and aldehyde dehydrogenase-1 (ALDH1), both known to be involved in HNSCC tumor development and progression (Chen et al., 2009; Wang et al., 2009; Clay et al., 2010; Bourguignon et al., 2012). These studies demonstrated that treatment of CD44v3^high^ALDH1^high^ HNSCC cells with matrix hyaluronan (HA) enhanced CSC-like properties, induced upregulation of DOT1L followed by H3K79 monomethylation on E-box elements located in promoter of miR-10b, resulting in the production of this miRNA (Ma et al., 2007). Moreover, it was shown that DOT1L participates in the upregulation of the cytoskeleton regulator RhoGTPase (RhoC) and of cIAP-2 and XIAP, belonging to the family of inhibitors of the apoptosis (IAPs), and facilitates invasion potential and cisplatin resistance of CD44v3^high^ALDH1^high^ cells (Bourguignon et al., 2016). In an additional study, amplification and upregulation of *MLLT3* gene was found in oral cavity squamous cell carcinoma (OSCC) cells, where this gene has been involved in cell migration and invasion in conjunction with DOT1L association and regulation of *CITED4* gene promoter and dysregulation of HIF-1α target genes *TWIST*, *MMP1*, *MMP2*, *VIM*, and *CDH1* (Wang C. I. et al., 2019).

Guo *et al.* reported high DOT1L levels in liver cancer, with respect to normal tissues, where its elevated expression is associated with the tumorigenic and self-renewal potential of HCC stem cells. Concerning the mechanism of DOT1L upregulation, it was shown that this epi-enzyme represents a target for miR-448, whose expression is activated by highly expressed SPDEF in normal liver cells. In cancer cells, this transcription factor becomes ubiquitinated and phosphorylated by CDK11B, which in turn is highly expressed in HCC, leading to proteasome-induced SPDEF degradation and downregulation of miR-448 expression (Guo et al., 2021). Taken together, these data show that the oncogenic and self-renewal potential of HCC stem cells is regulated by the SPDEF/miR-448/DOT1L axis (Guo et al., 2021).

In undifferentiated pleomorphic bone sarcoma (Ali et al., 2019) and in adrenocorticotropic hormone–independent macronodular adrenocortical hyperplasias (Cao et al., 2014) DOT1L recurrent mutations were identified, highlighting the potential role of deregulated chromatin remodeling pathways also in these cancer types.

The potential of DOT1L as a therapeutic target was also demonstrated for a subset of pancreatic adenocarcinomas, where high levels of the enzyme were found in 4.4% of 230 pancreatic tumors analyzed. Copy number variation analysis revealed that aberrant expression of this methyltransferase was not linked to the elevation of gene copy number implying that mechanisms other than this are involved (Loeser et al., 2017). In another study, Park et al. revealed DOT1L involvement in the development of resistance to PARP inhibitors (PARPi) in a pancreatic cancer model bearing BRCA2 mutation (Park et al., 2020). PARPi resistance was induced in this case by the amplification of a truncated BRCA2, whose depletion, or restoration of HR repair by increased RAD51 loading mediated by truncated BRCA2-DOT1L complex, restored cell sensitivity to olaparib and rucaparib (Park et al., 2020).

DOT1L involvement in the regulation of the antitumor immune response was described in pancreatic and colon cancers. These two cancer types are characterized by FOXM1 overexpression resulting from increased H3K79me2 methylation of its promoter (Zhou et al., 2019). Elevated expression and H3K79me2 methylation of FOXM1 were found also in bone marrow-derived dendritic cells (BMDCs) from tumor-bearing (TBM) and wild-type mice cultured with tumor-conditioned medium. Importantly, DOT1L inhibition induced BMDC maturation and functionality both *in vitro* and *in vivo*. Mechanistically, this was explained by the FOXM1-mediated activation of Wnt5a, an inhibitor of dendritic cells maturation (Bergenfelz et al., 2013), by binding to this gene promoter (Zhou et al., 2019).

Finally, a study of Liu et al. (2021) showed that DOT1L inhibition, combined with SHP2 blockade, represents an effective means for the treatment of a subset of KRAS-mutant cancers from different tissues. This was demonstrated by the application of the integrative analysis of phosphoproteome and drug sensitivity generated for a group of KRAS-mutant cancer cell lines with distinct biological, clinical, and therapeutic characteristics, representative of tumors with the poorest prognosis. In detail, combination of the two drugs resulted in synergetic inhibition of patient-derived xenograft growth, characterized by an acceptable toxicity level. Further analysis revealed that the observed beneficial effect was associated with DOT1L blockade-induced upregulation of PREX1, which in turn activated MAPK signaling and enhanced vulnerability of tumor cells to SHP2 inhibition (Liu et al., 2021).

Altogether, these data imply that DOT1L is involved in tumorigenesis and progression of several solid tumor types through context-dependent mechanisms that are now being in part clarified, although still requiring further investigation.

## DOT1 Pharmacological Inhibitors

**FIGURE 2:**
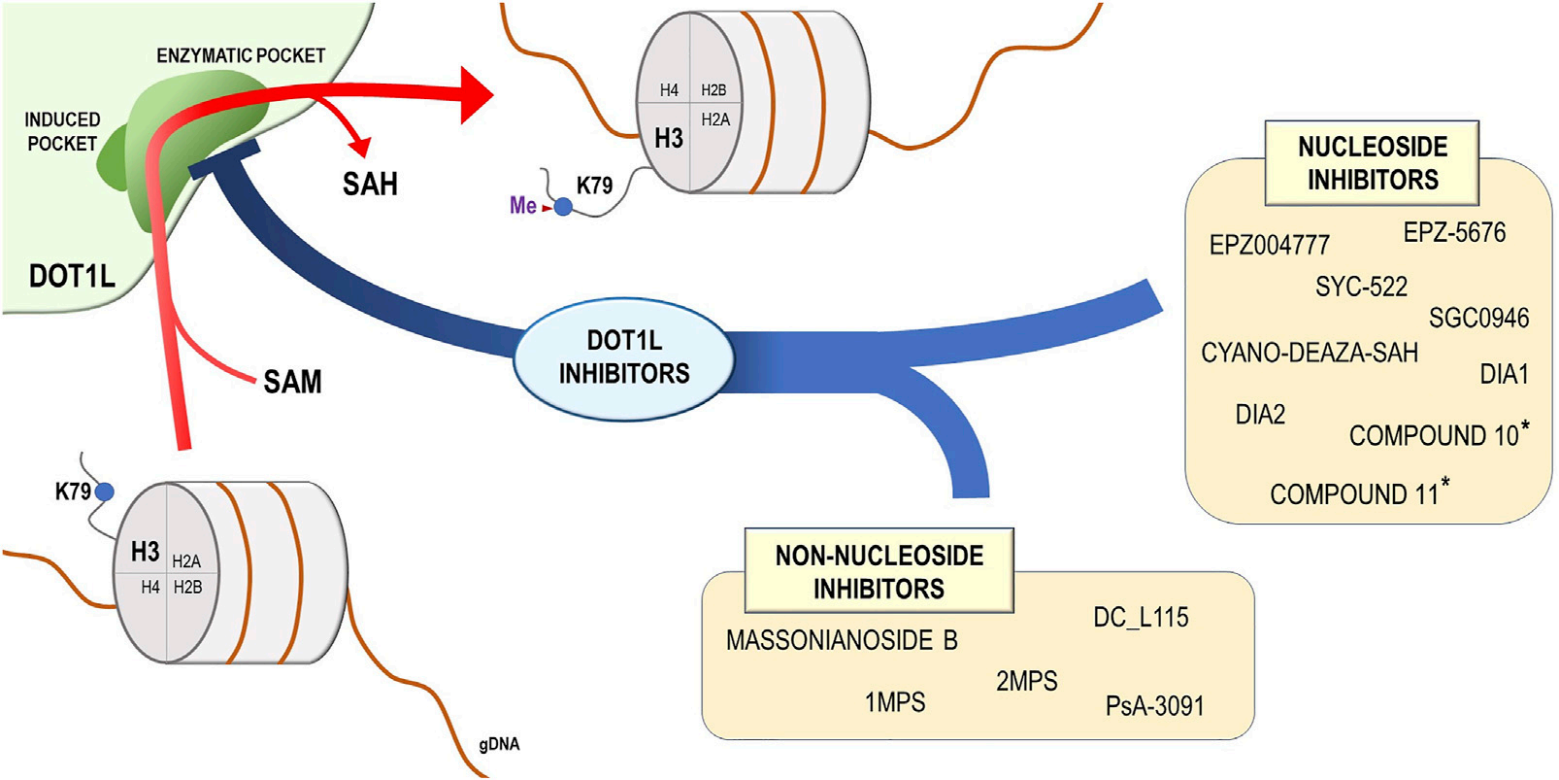
DOT1L mechanism of action and DOT1L inhibitors. DOT1L catalyzes the transfer of the methyl group from the S-adenosyl-L-methionine (SAM) to the lysine 79 residue of the histone H3 (H3K79), introducing a methyl group and releasing the S-adenosyl-L-homocysteine (SAH). On the basis of chemical structure, the DOT1L inhibitors are subdivided into two groups: nucleoside and non-nucleoside DOT1L small-molecule inhibitors. ^*^ These compounds were described in Perner et al. (2020).

The finding that DOT1L plays a key role in the development of MLL-rearranged leukemia-induced intense research in the field of drug discovery, aimed to design and synthesize small molecular inhibitors foreseeing blockade of DOT1L aberrant activity (Figure 2), caused by the acquired capability of MLL fusion proteins to interact with this methyltransferase (Okada et al., 2005; Bitoun et al., 2007; Mohan et al., 2010; Park et al., 2010). In a study of Daigle *et al.,* a mechanism-guided inhibitor discovery approach, exploiting the ability of this enzyme to bind and metabolize SAM, was applied to identify EPZ004777, an inhibitor characterized by a strong affinity for the SAM-binding pocket of the protein (Daigle et al., 2011). Coupled to a significant DOT1L specificity in comparison with other lysine methyltransferases, EPZ004777 showed a surprising ability to selectively inhibit proliferation of MLL-rearranged cells, decreasing H3K79 methylation level and inducing a profound transcriptome deregulation of cancer cells (Daigle et al., 2011).

Originally designed to hamper the progression of MLL-rearranged leukemia, a beneficial effect of EPZ004777 treatment was also observed for several solid malignancies, where DOT1L plays an important role. Colorectal cancer represents one of these examples, as far as EPZ004777 exposure of multiple CRC cell lines induced a marked reduction of cell viability and tumorigenicity, further confirmed using *in vivo* xenograft models (Yang et al., 2019). Moreover, this compound selectively inhibited colony formation ability and cell viability in AR-positive PCa cells compared with AR-negative ones, indirectly linking DOT1L oncogenic activity to AR status (Vatapalli et al., 2020), results confirmed after *in vivo* administration in xenograft mice models.

Despite the fact that EPZ004777 exerts a potent selective inhibitory effect on DOT1L activity, its *in vivo* effectiveness is limited by the poor pharmacokinetic properties of this compound, such as a very short plasma half-life that renders it not suitable for clinical use (Daigle et al., 2011).

To overcome this problem, Daigle *et al.* developed a novel DOT1L inhibitor, EPZ-5676, that retains the functional core and mechanism of action of EPZ004777 but shows a better inhibition potency and plasma half-life compared with its precursor, as well as improved selectivity and efficacy in reducing leukemic cell proliferation (Daigle et al., 2013). High intraperitoneal (IP) bioavailability and clearance of this molecule in animal models represented a fundamental improvement toward the clinical use of DOT1L inhibitors and induced a significant tumor regression in xenograft mice (Daigle et al., 2013). For this reason, Pinometostat (EPZ-5676 commercial name) has been primarily tested to treat relapsed/refractory leukemias bearing rearrangement of the MLL gene, and reached Phase 1 clinical trial (www.clinicaltrials.gov; NCT02141828). In a cohort study of 18 pediatric patients, Pinometostat had an acceptable safety profile and successfully inhibited DOT1L in leukemic blasts. Around 40% of treated individuals have benefited of a substantial reduction of peripheral or bone marrow blasts; however, no objective responses were observed (Shukla et al., 2016), indicating the requirement of further investigations. A second trial (www.clinicaltrials.gov; NCT01684150), performed on 51 adult MLL-r leukemia patients treated intravenously with Pinometostat for 28-day cycles at different doses, produced a complete remission in two patients, although the overall efficacy as a single agent was modest suggesting the need of further clinical investigations of combination approaches for leukemia treatment (Stein et al., 2018). At the moment, efficacy of Pinometostat in combination with standard chemotherapy is being evaluated in ongoing phase 1/2 clinical trial on both child and adult patients with newly diagnosed MLL-rearranged leukemia (www.clinicaltrials.gov; NCT03724084).

Recently, *in vitro* and *in vivo* translational studies have reported on EPZ-5676 efficacy for solid tumors treatment. In GC, cell lines highly expressing DOT1L showed a significant reduction of cell proliferation in the presence of EPZ-5676 (Song et al., 2020); moreover, mice inoculated with GC cells and treated with this compound developed smaller tumors compared with controls (Song et al., 2020). Interestingly hesperetin, a citrus flavanone present in citrus fruits, exhibited anti-tumorigenic effects in gastric cancer by reducing DOT1L stability and abundance, therefore affecting H3K79 methylation and the expression of cancer genes (Wang et al., 2021). Combined, these findings enforce the idea of a possible targeting of DOT1L with selective molecules to treat neoplasms of the gastrointestinal tract.

High levels of DOT1L were also detected in glioblastoma stem cells (Macleod et al., 2019). Moreover, this histone methyltransferase was found to be a fitness gene in seven GSC cultures (Macleod et al., 2019). In this context, EPZ-5676 treatment not only selectively inhibited GSCs growth, but also increased apoptosis and induced long-term morphological changes, decreasing cell stemness. Although the drug showed poor ability to pass through the blood-brain barrier, pretreatment of GSCs and subsequent inoculation in mice resulted in reduced tumor growth and increased cancer cell differentiation (Macleod et al., 2019). In lung cancer cells, TGF-β1-induced EMT is associated with decreased H3K79 methylation and EPZ-5676 treatment partially attenuated this transition in H358 cells by decreasing PD-L1 expression at protein level (Evanno et al., 2017). EPZ-5676 treatment for lung cancer appears to be, anyway, controversial, since most of the genes involved in EMT were not found to be affected by the drug, both in the presence and the absence of TGF-β1 stimulation (Evanno et al., 2017).

Recently, growing attention has been paid to DOT1L role in breast cancer and its possible targeting as a novel therapeutic approach. Starting from the observation that higher DOT1L levels strongly correlated with breast cancer progression, preliminary experiments indicated that its pharmacological inhibition by EPZ00477 and SYC-522, a potent new generation DOT1L inhibitor (Liu et al., 2014), reduced BC cell proliferation and induced cancer stem cells differentiation (Zhang et al., 2014). Moreover, noncanonical DOT1L inhibitors such as psammaplin A analogues induced similar effects in Triple-negative breast cancer cells by inhibition of cancer cell proliferation and metastasis (Byun et al., 2019). Importantly, Nassa *et al.* demonstrated that treatment with EPZ004777, EPZ-5676, and SGC0946, a recently synthetized DOT1L inhibitor, elicited significant effects in counteracting the oncogenic functions mediated by DOT1L-ERα interaction in hormone-responsive breast cancer (Nassa et al., 2019). The compounds showed marked efficacy also in endocrine therapy-resistant cells, suggesting that their antiestrogen-like responses could be exploited for treating patients who have acquired resistance to these drugs during hormonal therapies and therefore lack of valid alternatives (Nassa et al., 2019). These results point out that an important subset of BC, such as triple-negative or hormone-resistant ones, that still rely on adjuvant chemotherapy and suffer from extremely low patient survival rate, could benefit from treatment with DOT1L pharmacological inhibitors.

Moreover, compounds designed to repress DOT1L-mediated H3K79 methylation also showed efficacy in the treatment of ovarian cancer, further expanding the range of their potential applications for cancer therapy. Liu *et al.* observed that OC cells treatment with EPZ004777 and SGC0946 hampered C/EBPβ-mediated cisplatin resistance, abolishing the expression of key genes involved in this phenomenon (Liu D. et al., 2018). Similarly, Salvati *et al.* demonstrated that DOT1L inhibitors EPZ004777, EPZ-5676, and SGC0946 were able to counteract DOT1L oncogenic functions of ERα-positive OC, causing cell cycle progression delay together with the reduction of cell proliferation and colony formation (Salvati et al., 2019).

Finally, DOT1L inhibitors showed effects also in reducing retinoblastoma cells aggressiveness, where they enhanced sensitivity to chemotherapy and affected cell proliferation (Mao et al., 2021). Moreover, combination therapy with DOT1L and SHP2 inhibitors was demonstrated to be effective in treating a specific subset of KRAS-mutant cancers characterized by a very poor prognosis (Liu et al., 2021).

Although the encouraging results achieved from multiple studies are pushing toward further development of DOT1L-targeting drugs, there are still some major issues that necessarily need to be addressed, such as off-targeting and consequent side toxicity. In this regard, Lillico *et al.* analyzed the effects of pharmacological inhibition of DOT1L, LSD1, and HDAC on the molecular phenotype of MLL-AF9 rearranged cell line MOLM-13, with particular attention on the changes that these agents produce on the global and residue-specific histone posttranslational modifications profile (Lillico et al., 2018). The authors confirmed that EPZ-5676 was indeed the most effective in reducing HOXA9 expression among the tested compounds, but they also surprisingly observed that its administration produced a global increase in histone demethylation, in paradoxical opposition to expected results. This effect was due to EPZ-mediated, time-dependent, downregulation of LSD1 expression, which therefore did not rely on aspecific binding of EPZ-5676 to LSD1 protein. They also identified a general decrease in many histone H3 modifications induced by EPZ and correlated this phenomenon to downregulation of genes from most classes of histone-modifying enzymes (Lillico et al., 2018). Hence, even considering that EPZ-5676 secondary effects on LSD1 enforce its inhibitory activities on MLL-rearranged leukemia cells, it is noteworthy that the extent of off-target consequences of DOT1L-targeting can produce substantial increase in drug toxicity, since most of the epigenetic modifications affected are crucial for normal cells biology. This considerably impacts the efficacy of these compounds, delaying their use in clinical practice.

Of concern, the relevance of DOT1L for cell development results in most of the adverse effects encountered during EPZ-5676 clinical trials. In particular, as described by Shukla et al. (2016) and Stein et al. (2018), leukemia treatment with Pinometostat can produce relevant toxicity at the bone marrow level, leading to pathogenic reduction of large part of blood elements progenitors and consequent febrile neutropenia, anemia, leukopenia, thrombocytopenia, and lymphopenia.

Despite the necessary improvements required to ameliorate for DOT1L-targeting molecules to improve their overall safety profile, the aforementioned results indicate that DOT1L oncogenic potential lays beyond leukemia and embraces a number of solid cancers that potentially could benefit from DOT1L pharmacological inhibition.

## Novel DOT1 Inhibitors as a Potential Therapeutic Agents for Solid Tumors

The multiple evidences provided for the efficacy of DOT1L inhibitors for both leukemia and solid tumors treatment were inducing a growth of interest in developing novel compounds with improved inhibitory potential and bioavailability (Figure 2).

Although most of the commercially available DOT1L-selective inhibitors, such as EPZ004777 and EPZ5-5676, compete with the methyl-donating cofactor SAM and lock the SAM-binding pocket (Basavapathruni et al., 2012), there are other molecules, able to induce a similar DOT1L inhibitory effect by partially engaging the catalytic site (Chen C. et al., 2016; Scheufler et al., 2016), suggesting that multiple sites of DOT1L can be targeted to hamper its activity. In this context, Yu *et al.* modified the adenosine ring of S-adenosyl-L-homocysteine (SAH), a SAM competitive inhibitor, with halogen atom and observed that this compound is able to selectively bind DOT1L hydrophobic cavity near the adenosine-binding site (Yu et al., 2013). Although strongly selective, the halogen group induced in the generated compounds a poor cell penetration and therefore limited their *in vitro* and *in vivo* application. Thus, Spurr and colleagues moved toward the design of new nucleoside derivatives as substitutes, still able to bind to the hydrophobic site. They observed that incorporation of an alternative polar nitrile instead of the halogen could produce an effective DOT1L inhibition. Moreover, they identified and characterized cyano-deaza-SAH, whose polar 5-nitrile strongly bound the hydrophobic pocket of DOT1L, opening the way for its further evaluation in cell-based assays (Spurr et al., 2016). Möbitz *et al.*, on the other hand, extended their focus on the identification of molecules also able to interact with the adenosine-binding pocket itself by applying fragment linking to increase inhibitors potential by creating multiple biochemically active fragments connections (Möbitz et al., 2017). Their study led to the discovery of a molecule, named 2, which mimicked SAM binding to DOT1L in the adenosine pocket and that, when linked to a previously identified compound targeting the induced cavity (named 3), delivered a new DOT1L inhibitor (named 7) which showed high DOT1L selectivity combined to potency (Möbitz et al., 2017).

To overcome the limitations provided by fragment-based screenings for the identification of novel DOT1L inhibitors, Song *et al.* developed a high-throughput screening program based on Amplified Luminescent Proximity Homogeneous Assay (ALPHA) LISA assay to directly test the ability of a large library of compounds in modulating DOT1L activity through the measurement of H3K79me2 levels (Song et al., 2018). This method led to the identification of a subset of molecules, structurally similar among them, with enhanced DOT1L binding and inhibitory potential, as well as ability to induce effective inhibition of MV4-11 leukemia cells proliferation, probably through induction of cell cycle arrest and apoptosis (Song et al., 2018).

Recently, Perner *et al.* reported the preclinical evaluation of two SAM-competitive DOT1L-targeting agents, namely compounds 10 and 11, which displayed improved pharmacokinetics properties when compared with EPZ-5676 and potent antileukemic activity (Perner et al., 2020). The authors observed that these molecules, other than showing selectivity for MLL-rearranged leukemia cells and a sensitivity profile similar to Pinometostat, were able to induce differentiation in murine MLL-AF9 leukemia cells and efficiently modulate the expression of MLL-AF9 target genes. Moreover, treatment of patient-derived xenograft (PDX) models of MLL-rearranged leukemia with 75 mg/kg twice daily of both compounds resulted in a significant reduction of leukemia burden in bone marrow, spleen, and peripheral blood after 4 weeks, accompanied by a parallel good tolerance profile (Perner et al., 2020).

In comparison with nucleoside compounds, which inhibitory effect mainly relies on the interaction with the SAM/SAH-binding pocket, non-nucleoside molecules target different DOT1L regions. One of these compounds has been described in a study of Chen *et al.*, who applied virtual screening on the SPECS database and identified a set of appealing potential DOT1L inhibitors, among which DC_L115 demonstrated an outstanding potency, possessed a structure, that does not resemble SAM scaffold, and was able to selectively inhibit MLL-rearranged cell proliferation (Chen S. et al., 2016). These effects were further enhanced by the addiction of an amino side chain to the compound, a modification that increased its ability to pass cell membrane and penetrate cancer cells (Zhang et al., 2018). Consequently, Sabatini *et al.* combined different ligand-based and structure-based computational methods to screen a set of known SAM-competitive DOT1L inhibitors and created a predictive model to apply to a set of molecules with unknown DOT1L-binding potential. Among them, the authors identified two compounds, named 1MPS and 2MPS, which showed inhibitory abilities of the enzyme at micromolar concentrations. In addition, 2MPS showed the ability to bind to DOT1L in a completely different site compared with known inhibitors, presenting new insights toward the development of non-nucleoside compounds (Sabatino et al., 2018).

Using a pharmacophore-based virtual screening of a chemical library, Chen *et al.* identified Massonianoside B (MA), a natural product with DOT1L inhibitory potential, which retained a noncanonical, non-nucleoside structure. This molecule showed high selectivity and the ability to reduce the level of H3K79 mono- and di-methylation in a dose-dependent manner, while also causing apoptosis increase in MLL-rearranged leukemia cell and deregulation of known DOT1L target genes such as *HOXA9* and *MEIS1*, presenting a novel, highly potential, scaffold to exploit for DOT1L inhibitors development (Chen and Park, 2019). Soon after, Stauffer *et al.* designed a series of molecules able to inhibit DOT1L activity by binding to the induced cavity near the SAM-binding pocket, but with a mutually exclusive trend, leading to an apparent SAM competitive inhibition (Stauffer et al., 2019). Although the authors managed to achieve better pharmacokinetic properties after subcutaneous administration for the new compounds in rodent models, they still struggled to achieve acceptable efficacy *in vivo* due to the difficulties linked to DOT1L drugs development (Stauffer et al., 2019).

Interestingly, Gibbons *et al.* applied structure-based virtual screening, followed by synthesis, biological activity, and molecular modeling studies to identify and develop a non-adenosine series of DOT1L inhibitors. Although incorporation of a bulky hydrophobic group led to the discovery of a subgroup of inhibitors with improved potency, they still retained a modest potential, and were therefore intended to serve as scaffold for further optimization of original non-nucleoside inhibitors (Gibbons et al., 2020). Finally, in a recent work, Bon *et al.* reported the synthesis of two compounds, Dia1 and Dia2, analogs of EPZ004777, differentiated by the presence of a chiral carbon in their structure (Bon et al., 2021). Among the two diastereoisomers, Dia2 showed a much stronger ability at inhibiting the enzymatic activity of DOT1L *in vitro*, while also presenting an increased metabolic stability in mouse liver microsomes, even if compared with the widely used EPZ-5676. These effects were accompanied by inhibition of MLLr leukemia cells proliferation after long treatment with low doses of the compound, mainly through cytostatic mechanisms. The lack of cytotoxicity, which instead characterizes EPZ-5676 treatment, renders Dia2 an optimal chemical compound for investigation of long-term consequences of DOT1L inhibition (Bon et al., 2021).

However, identification of novel molecules may help overcome the problems of the existing DOT1L-targeting drugs and provide novel tools for epigenetic modifications-targeting therapies.

## Conclusion

Development and progression of solid tumors depend upon complex biological events still not fully characterized. Among them, epigenetic regulation of gene expression through histone modifications, histone methylation in particular, covers a pivotal role being involved, among others, in fine tuning of cell proliferation and differentiation, mediated by specific controls of transcriptional elongation, DNA recombination, and damage repair. DOT1L catalyzes lysine 79 methylation in H3 histone and is essential in many aspects of cancer cell biology, including genome integrity.

Multiple studies, summarized here, focused on the characterization of DOT1L actions in solid tumors, revealing multiple molecular mechanisms underlying a key involvement of this epigenetic regulator and its potential as a promising new actionable target for new therapeutic regimens against these diseases. As a consequence, there has been an increasing interest of the scientific community toward developing novel small-molecule inhibitors of this HMT, leading to new drugs showing great efficacy *in vitro* and *in vivo*, in animal models. Progress in this field, however, has been slowed down so far by a relative absence of structural diversity of these compounds and their poor bioavailability, which both represent an obstacle for their clinical development. Indeed, major challenges remain to be solved before the introduction of DOT1L inhibitors in clinical practice, in particular for solid tumor therapy. Among these, it is worth mentioning their modest activity when administrated as single agents, needing a further improvement of possible combinatorial therapies. To date, only EPZ-5676 has entered clinical trials, making the market of DOT1L-targeted cancer treatment essentially vacant. Based on these considerations, continuous design and improvement of new scaffold molecules targeting DOT1L are still sought after, since they held the promise to be exploitable not only against leukemias, where they were first studied, but also for treatment of solid cancers, where as summarized in this review this enzyme has been shown to be a key regulator of several basic mechanisms controlling carcinogenesis, tumor growth, and metastatization.
